# Advances in 3D bioprinting for modeling the blood-brain barrier in neurodegenerative diseases

**DOI:** 10.3389/fmolb.2025.1703403

**Published:** 2025-11-13

**Authors:** Shirleen Tan, Lifeng Qiu, Jolene Wei Ling Lee, Li Zeng

**Affiliations:** 1 Neural Stem Cell Research Lab, Research Department, National Neuroscience Institute, Singapore, Singapore; 2 Neuroscience and Behavioral Disorders Program, DUKE-NUS Graduate Medical School, Singapore, Singapore; 3 Centre for Molecular Neuropathology, Lee Kong Chian School of Medicine, Nanyang Technology University, Singapore, Singapore

**Keywords:** blood-brain barrier, 3D bioprinting, neurodegenerative diseases, bioink, disease modeling

## Abstract

The growing burden of neurodegenerative diseases (NDD) on healthcare systems, driven by global aging population, has increased interest in modelling the blood-brain barrier (BBB). While microfluidic platforms have been widely used to model the BBB, they remain limited by complex fabrication techniques, low-throughput, and restricted control over BBB geometry. Recent advancements in three-dimensional (3D) bioprinting offer promising strategies to overcome these constraints and to enable the generation of physiologically relevant BBB models. This review examines the recent progress in 3D bioprinting approaches to model human *in vitro* BBB, with a focus on their applications in NDD research. We first summarise current 3D bioprinting techniques and strategies, including the selection of bioinks and geometry design. Subsequently, we address the evaluation methods for *in vitro* BBB modelling and their relevance to disease modelling. Finally, we identify key challenges and future directions aimed at improving resolution, reproducibility, and functional 3D-printed BBB constructs for use in NDD modelling and drug development.

## 1 Introduction

Neurodegenerative diseases (NDD), including Alzheimer’s disease (AD) and Parkinson’s disease (PD), affect approximately 15% of the global population and pose a growing challenge to healthcare systems worldwide (Liu et al., 2022). A critical factor in NDD pathogenesis is the dysfunction of the blood-brain barrier (BBB) (Sweeney et al., 2018; Tran et al., 2022), which plays a central role in maintaining central nervous system (CNS) homeostasis by tightly regulating molecular exchange between the bloodstream and the brain (Kadry et al., 2020; Segarra et al., 2021). The BBB protects neural tissue from neurotoxic plasma components, blood cells, and pathogens, while ensuring optimal neuronal function.

Evidence suggests that disruption of BBB, leading to the loss of selective permeability, may precede and contribute to neuronal degeneration by allowing the entry of neurotoxic plasma components, inflammatory mediators, and immune cells into the CNS (Bell et al., 2010; Takata, 2021). This breach can amplify neuroinflammation and impair clearance of pathological proteins such as accumulated amyloid-beta (Aβ) in AD (Mawuenyega et al., 2010) and misfolded alpha-synuclein in PD (Stefanis et al., 2012). Understanding the mechanisms underlying BBB dysfunction and evaluating strategies to restore barrier integrity are therefore essential for advancing NDD research and therapy development.

Modelling the BBB has thus become essential for elucidating NDD pathogenesis and evaluating CNS-targeted therapies. However, due to the restrictive permeability and structural complexity of the human BBB, accurate modelling remains challenging. Traditional modelling approaches include the two-dimensional (2D) Transwell co-culture platform, which typically involves endothelial cells (EC) on the apical surface (representing the blood side) and other supporting cells on the basolateral surface (representing the brain side). The two compartments are separated by a porous membrane which allows molecular exchange and intercellular communication (Stone et al., 2019).

Although the 2D Transwell system has provided valuable insights, it is limited in its ability to recapitulate spatial organization, dynamic flow conditions and microenvironment of the human BBB (Yan et al., 2011). Furthermore, animal models often fail to fully recapitulate human BBB physiology, limiting their translational relevance (Badawi et al., 2024; Helman et al., 2016). These limitations have therefore driven the development of more advanced systems that better mimic native human BBB physiology.

Advanced technologies such as microfluidic organ-on-a-chip platforms and stem cell-derived BBB models have emerged in response. Among these, three-dimensional (3D) bioprinting stands out for its ability to generate spatially organized, customizable, and physiologically relevant *in vitro* BBB constructs. By integrating vascular geometry, multicellular interactions, extracellular matrix (ECM) composition, and perfusable flow, 3D bioprinting provides a powerful platform for studying BBB dysfunction in NDD and accelerating CNS-targeted drug development.

**FIGURE 1:**
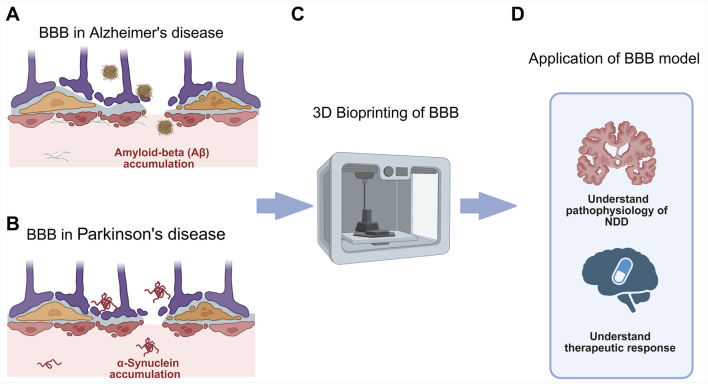
**(A)** BBB dysfunction in AD which involves accumulation of amyloid plaque. **(B)** BBB dysfunction in PD which involves accumulation of misfolded alpha-synuclein protein. **(C)** 3D bioprinting to create *in vitro* models of the BBB in NDD. **(D)** Application of BBB model in NDD research, to elucidate NDD pathophysiology for identification of novel therapeutic targets and therapy development via high-throughput drug screening.

In this review, we explore the use of 3D bioprinting for modelling human BBB, with a focus on applications in NDD research (Figure 1). We begin by outlining the anatomical and functional features of the BBB, followed by key design considerations for *in vitro* models. We then compare existing modelling approaches while emphasising the advantages of 3D bioprinting. Subsequently, we discuss bioprinting techniques, bioink optimisation, and geometric design strategies, and conclude with functional assessment methods and the potential application of bioprinted BBB models in NDD research.

### 1.1 Anatomy of the BBB

**FIGURE 2:**
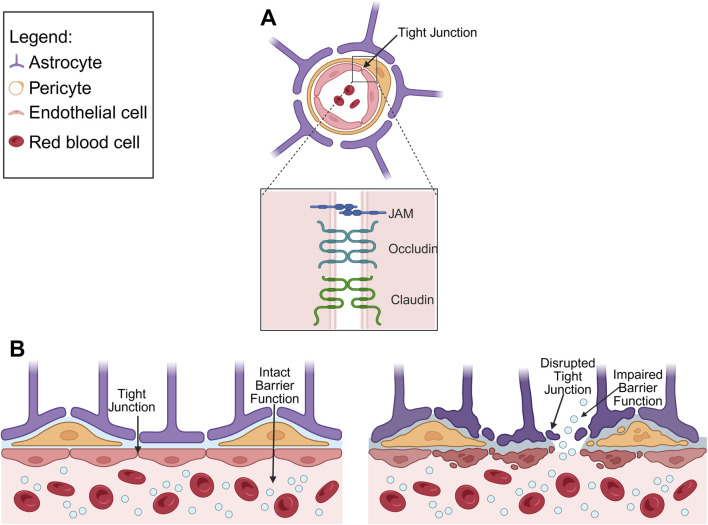
**(A)** Schematic diagram depicting anatomy of the healthy BBB, highlighting TJ proteins (junctional adhesion molecule (JAM), occludin and claudin). **(B)** Comparison of healthy BBB and diseased BBB, illustrating disrupted TJ and impaired barrier function.

Accurate BBB modelling requires replication of both its cellular composition and the structure of the surrounding ECM. The main cellular components of the BBB include brain microvascular endothelial cells (BMEC), pericytes (PC), astrocytes (AC) (Figure 2). BMEC differ markedly from EC in peripheral tissues. They exhibit low rate of transcytosis and are interconnected by complex tight junctions (TJ) that restrict paracellular flux and diffusion (Rubin et al., 1999).

PC, embedded within the basement membrane, regulate BBB permeability by modulating the expression of TJ and adherent junction proteins in BMEC, thereby influencing barrier tightness and vascular stability (Sweeney et al., 2016; Sweeney et al., 2019). AC contribute to the regulation of BBB permeability through their end-feet, which envelop the blood vessels and form close physical interactions with BMEC. These astrocytic processes also respond to CNS injury and help maintain homeostasis (Pekny et al., 2016). The BBB ECM differs from other tissues, lacking hyaluronic acid (HA) and consisting primarily of collagen IV, laminin, nidogen, perlecan and fibronectin (Reed et al., 2019). These components provide both structural support and biochemical cues that influence BBB cellular behaviour and barrier function.

TJ, which are fundamental to BBB integrity, comprise of membrane proteins including occludin, claudins and JAM (Stamatovic et al., 2016). Occludin forms oligomers that regulate solute diffusion across the TJ that can be disrupted under pathological conditions such as hypoxia-regeneration (Lochhead et al., 2010). Claudins, a family of tetraspan transmembrane proteins, determine the tissue, size, and charge properties of the TJ (Krause et al., 2008). Among these, claudin-3 and claudin-5 are particular important for maintaining BBB integrity, and reduced claudin-5 expression increased barrier permeability (Luissint et al., 2012). JAMs, members of the immunoglobulin superfamily, regulate TJ assembly through interactions with cell polarity related proteins, thereby reducing permeability (Hudson et al., 2021). Notably, JAM-1 is involved in the early stages of TJ formation and is essential for BBB integrity (Jia et al., 2013).

### 1.2 Key design considerations for *in vitro* BBB modelling

**FIGURE 3:**
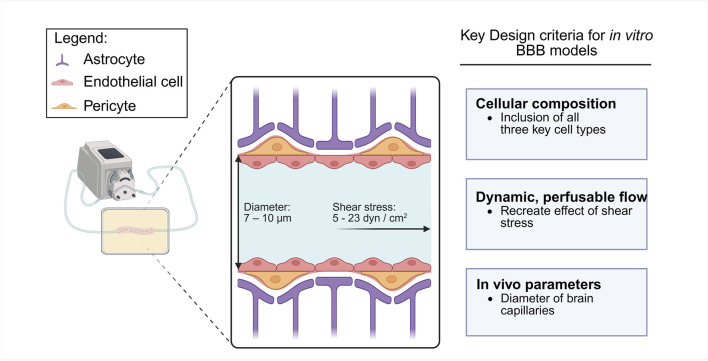
Schematic diagram of key design considerations for *in vitro* BBB modelling. Key design criteria include: 1) cellular composition consisting of AC, EC and PC, 2) a dynamic and perfusable flow system with physiological shear stress, and 3) physical parameters similar to *in vivo* brain capillaries.

Physiologically relevant BBB models aim to replicate *in vivo* function as closely as possible, encompassing appropriate cellular composition and structural integrity. Key design considerations are summarised in Figure 3. One of the most critical aspects is the inclusion of all three key BBB cell types–BMEC, AC and PC (Jamieson et al., 2017). Incorporating these cells enhances TJ formation and barrier tightness, allowing more accurate replication of the anatomical and functional complexity of the BBB (Vetter et al., 2025). Beyond cellular composition, model reproducibility and homogeneity are also crucial considerations when developing NDD specific BBB models to ensure consistent and reliable disease modelling (Winkelman et al., 2021). Variability in cell sourcing, culture conditions, or scaffold composition can significantly impact barrier properties and reduce translational relevance.

Another important criterion is the inclusion of dynamic, perfusable flow (Bolden et al., 2023; Potjewyd et al., 2021). Perfusable models that simulate capillary blood flow recreate shear stress experienced by EC *in vivo* that is reported to be between 5 and 23 dyn/cm^2^ in human brain capillaries (Wang et al., 2020). This mechanical stimulus influences cell alignment, morphology, and upregulation of TJ proteins which are key elements for maintaining barrier integrity and function (Yue et al., 2020). By integrating controlled flow conditions, *in vitro* BBB systems can better emulate the native microenvironment, enabling the study of vascular contributions to NDD pathogenesis and for evaluating therapeutic strategies.

Finally, *in vitro* BBB models should strive to reproduce *in vivo* physiological parameters. High-resolution fabrication is required to achieve structural features comparable to brain capillaries, which are 7–10 μm in diameter (Pandey et al., 2016; Wong et al., 2013). Another key metric is the trans-epithelial electrical resistance (TEER), which reflects barrier integrity; physiologically relevant models should aim for *in vivo* TEER values ranging from 1,500 to 8,000 Ωcm^2^ (Crone et al., 1982; Reichel et al., 2003; Wolff et al., 2015).

### 1.3 Current *in vitro* BBB modelling approaches

Various techniques have been developed to construct *in vitro* BBB models, including microfluidics and 3D bioprinting (Table 1). Among these, microfluidic approaches are currently more prevalent in the literature, in part due to their ability to incorporate dynamic flow and mimic physiological shear-stress conditions (Jagtiani et al., 2022).

**TABLE 1:** Advantages and disadvantages of microfluidic against 3D bioprinting methods for BBB modelling.

Model type	Advantages	Disadvantages	References
Microfluidic BBB	Shear stress incorporation, long-term viability	Complex fabrication, stiffness mismatch	Royse, 2024; Wei (2023)
3D bioprinted BBB	High resolution, reproducible, customisable geometry	Resolution variability by method, vascularization challenge	Galpayage Dona, 2023; Yue (2020)

Microfluidic models enable perfusion, allowing the development of dynamic BBB models with improved barrier tightness and functionality. EC cultured under flow conditions showed elongated cell morphology and higher localisation of TJ proteins which are features associated with enhanced barrier integrity (Wei et al., 2023).

Additionally, perfusion also supports cell viability by facilitating metabolite and nutrient diffusion, promoting long-term culture maintenance. However, stiff materials used in microfluidic devices can alter mechanotransduction signalling due to stiffness mismatches with native tissue (Potjewyd et al., 2021). Furthermore, complex fabrication procedures and small construct dimensions can limit meaningful multicellular interactions, which are essential for replicating the BBB multicellular nature (Royse et al., 2024).

To address these challenges, 3D bioprinting has recently been integrated with microfluidic devices, offering a promising hybrid approach. 3D bioprinting enables spatially controlled deposition of multiple cell types and ECM components, supporting the creation of high-resolution, reproducible and customisable models (Tang et al., 2021; Yue et al., 2020). Galpayage Dona et al. demonstrated the use of digital light processing (DLP)-based bioprinting to encapsulated human AC within a vascular lumen surrounded by PC and primary human BMEC, successfully generating a perfusable microvascular network that replicated key BBB features (Galpayage Dona et al., 2023). While microfluidics currently dominates the field, 3D bioprinting offers superior architectural control and scalability, making it a promising platform for next-generation BBB models.

## 2 3D bioprinting strategies for BBB modelling

3D bioprinting utilises computer aided design models to fabricate precise 3D structures. These models can be developed from medical imaging data such as radiological images, allowing for the recreation of anatomically accurate tissue architectures. When combined with chemical crosslinking, 3D bioprinting can generate high-resolution, multicellular structures that closely mimic native tissue environments (Potjewyd et al., 2021). Importantly, this technique enables reproducible and consistent manufacturing of *in vitro* models (Jagtiani et al., 2022), allowing for better standardization and comparability across studies.

### 2.1 Bioprinting techniques for BBB fabrication

**FIGURE 4:**
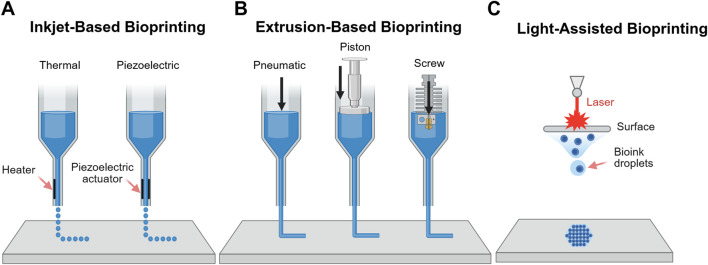
Schematic representation of 3D bioprinting methods. **(A)** Inkjet-based bioprinting which operates via thermal or piezoelectric mechanisms **(B)** Extrusion-based bioprinting controlled via pneumatic, piston or screw systems; and **(C)** LAP.

Three major categories of 3D bioprinting technologies are commonly employed in tissue engineering applications: inkjet-based, extrusion-based and light-assisted printing (LAP) methods (Cho et al., 2019) (Figure 4). Inkjet-based bioprinting involves the deposition of controlled volumes of bioink at predefined locations, either through thermal inkjet bioprinting or piezoelectric inkjet bioprinting, which differ in how they overcome surface tension to eject bioink droplets from the nozzle. Although inkjet bioprinting allows fabrication of complex tissue constructs with different compositions and is both affordable and versatile, its use in BBB modelling is limited by difficulties in generating porous, tissue-like constructs and the requirement for low-viscosity bioinks, which restricts material choices (Gudapati et al., 2016).

Extrusion-based bioprinting deposits continuous filaments of biomaterial through a nozzle, controlled pneumatically or mechanically. This approach accommodates a broader range of bioink viscosities and supports very high cell densities. However, it is limited by lower resolution compared to other methods, the risk of nozzle clogging and reduced cell viability due to shear stress during extrusion (Hölzl et al., 2016). Notably, co-axial extrusion enables the fabrication of hollow fibres that mimic capillary geometry, making it particularly promising for modelling BBB (Mohan TS et al., 2022).

LAP methods, such as DLP and two-photon polymerisation techniques, offer precise control over material properties and high-resolution printing (Galpayage Dona et al., 2023). As these methods are nozzle-free, they eliminate shear stress on cells during printing, preserving cell viability (Hölzl et al., 2016). Although there are concerns over cytotoxicity from photo-crosslinking (Mironi-Harpaz et al., 2012; parhi, 2017), multiple studies have shown that these effects are minimal, with no significant impact on cell viability (Galpayage Dona et al., 2023; Haring et al., 2019). Among the available technologies, LAP methods are currently the most widely used for BBB bioprinting due to their superior resolution and precision.

### 2.2 Bioink development and optimization

Selecting an appropriate bioink is crucial in 3D bioprinting to recapitulate the BBB complex architecture and function. Ideal bioinks must fulfil criteria such as biocompatibility, printability, mechanical integrity, and the ability to support BBB-specific cellular functions. Bioinks are typically categorized as either natural or synthetic.

Natural bioinks, such as HA, collagen, gelatin, alginate, and Matrigel, provide intrinsic biological cues but often lack mechanical robustness. HA supports cell migration and proliferation (Potjewyd et al., 2018) but exhibits poor mechanical strength, requiring combination with other polymers to enhance structural stability and printability (Tang et al., 2021). Collagen, particularly type IV, is a native component of the BBB ECM, offering high bioactivity through Arginine-Glycine-Aspartic (RGD) motifs (Hölzl et al., 2016; Tang et al., 2021). However, its slow gelation and low stiffness restrict independent use, requiring reinforcement with additional agents (Potjewyd et al., 2018). Gelatin, a hydrolysed form of collagen, retains bioactive domains (Asim et al., 2023) but lacks photo-crosslinkable groups. This limitation can be overcome by chemical modification (e.g., methacrylamide or thiol-ene functionalisation), which enhances print fidelity and reproducibility (Dobos et al., 2021). Gelatin also enables modular designs that permit post-printing dissolution and tissue remodelling, making it suitable for soft-tissue BBB constructs (Jagtiani et al., 2022). A notable application is its combination with fibrinogen for the coculture of BMEC, AC, and PC, which improved cell morphology compared with conventional 2D cultures (Tung et al., 2024). Alginate, derived from brown algae, undergoes rapid ionic crosslinking with calcium, allowing physiological gelation while maintaining cell viability. Although rigidity and porosity are calcium concentration dependent, no adverse effects on cell morphology or function were reported (Oh et al., 2023). Blending alginate with low viscosity collagen yields a compliant bioink suitable for mimicking native BBB tissue (Potjewyd et al., 2021). Lastly, Matrigel, a thermosensitive ECM extract rich in laminin and collagen IV, supports differentiation and barrier formation (Oh et al., 2023; Tang et al., 2021). Nonetheless, its murine origin and batch variability compromise reproducibility and limit clinical translation.

Synthetic bioinks such as polyethylene glycol (PEG) provide well-controlled mechanical properties and reproducible performance. When functionalised as PEG-diacrylate (PEGDA) or PEG-norbornene, PEG enables photo-crosslinking, allowing precise spatial patterning and reducing cell death during live-cell printing (Gudapati et al., 2016; Paone et al., 2024). However, PEG lacks inherent bioactivity and therefore requires modification with ECM-derived peptides to promote cell-material interactions. Functional motifs such as RGD and Isoleucine-Lysine-Valine-Alanine-Valine (IKVAV) facilitate cell adhesion and spreading (Matthiesen et al., 2023), while Histidine-Alanine-Valine-Aspartate-Isoleucine (HAVDI) supports endothelial monolayer formation and TJ assembly, as evidenced by increased localization of zonula occludens-1 (ZO-1) even in the absence of flow (Paone et al., 2024). Incorporating these bioactive peptides into PEG-based inks preserves mechanical stability while substantially enhancing biological functionality.

As summarized in Table 2, natural bioinks excel in cell-matrix interactions but often require mechanical reinforcement, whereas synthetic bioinks are structurally tuneable yet need biofunctionalisation for physiological relevance. Current challenges include improving reproducibility, vascularisation, long-term stability, and scalability of the 3D bioinks. To address these issues, hybrid bioinks combining natural and synthetic components, enhanced with cell-instructive peptides, is a promising strategy. Future directions should prioritise advanced crosslinking strategies, peptide-based customization and biofunctionalization, standardized formulations, and validation in perfused, shear-responsive systems to better model BBB physiology and improve translational relevance.

**TABLE 2:** Summary of bioinks suitable for 3D BBB bioprinting.

Bioink	Gelation mechanism	Cell adhesion	Advantages	Limitations	References
HA	Photo-crosslink	Inherent	Promote cell migration and proliferation	Poor mechanical properties	Potjewyd et al. (2018), Tang et al. (2021)
Collagen	Thermal/pH	Inherent	High porosity	Slow gelation	Hölzl et al. (2016), Tang et al. (2021), Wang et al. (2023)
Gelatin	Thermal	Inherent	Good rheology/thermally responsive	Affect cell viability	Oh et al. (2023), Tang et al. (2021)
Alginate	Calcium ions	Chemical modification	Fast gelation	Lack cell adhesion peptides	Potjewyd and Hooper (2021), Wang et al. (2023)
Matrigel	Thermal	Inherent	Similar to vascular ECM	Animal origin/batch variation	Oh et al. (2023), Potjewyd and Hopper (2021), Tang (2021)
PEG	Photo- crosslink	Chemical modification	Biocompatible/tuneable mechanical properties	Low optical transparency with high concentration	Galpayage Dona et al. (2023), Gudapati et al. (2016), Tang et al. (2021)

### 2.3 Geometry design considerations

In addition to the choice of bioinks, the geometrical fidelity is critical for BBB modelling. Ideally, the model should closely replicate the dimensions and structure of microvascular capillaries, which vary in diameter according to the anatomical location of the microvessel (DeStefano et al., 2018). Accurately mimicking these capillary dimensions is essential for reproducing physiological shear stress and cellular organisation. However, achieving such high-resolution features with direct bioprinting methods can be technically challenging. In contrast, high-resolution bioprinting methods, such as the biomimetic model developed by Marino A. et al. Achieved resolutions similar to that of the *in vivo* dimensions using two photon lithography (TPL) (Marino et al., 2018).

Beyond resolution, the model’s architectural design should accurately reflect the cylindrical geometry of the native microvessel. To achieve this, indirect bioprinting methods which incorporates a removable sacrificial biomaterial have been used in order to create cylindrical channels that can be subsequently seeded with EC, thereby ensuring a physiologically relevant structure (Potjewyd et al., 2018). This method allows for the fabrication of perfusable, physiologically relevant constructs that support cellular alignment and barrier formation under flow conditions.

## 3 Functional assessment of BBB models

**FIGURE 5:**
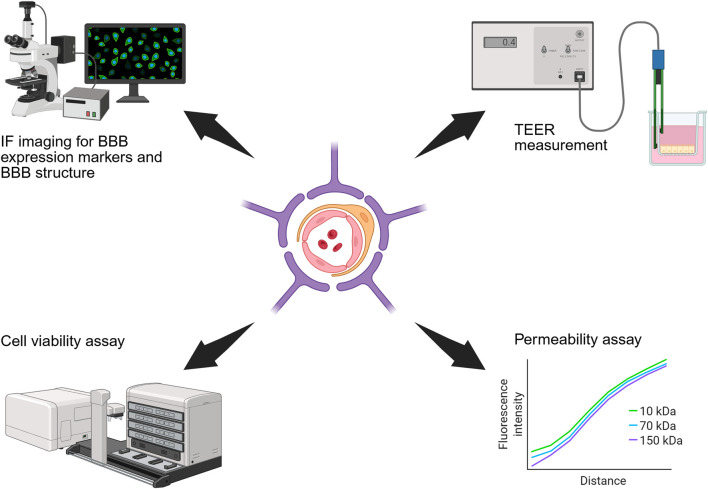
Schematic diagram of methods for functional assessment of BBB models. These include IF staining for BBB specific markers, TEER measurement, cell viability assay, and permeability assays.

Rigorous functional assessment is essential to validate *in vitro* BBB models and confirm they capture the physiological and pathological features of the native BBB. As illustrated in Figure 5, the functionality of *in vitro* BBB models can be evaluated based on integrity, permeability, cellular function and key molecular expression (DeStefano et al., 2018).

BBB integrity is commonly assessed by immunofluorescence (IF) staining of TJ proteins (ZO-1, claudin-5, JAMs) and efflux transporters (e.g., P-glycoprotein) which are indicative of barrier formation (Langen et al., 2019). TEER remains the gold standard for non-destructive, real-time assessment of barrier tightness, although electrode placement and opacity can introduce variability (Srinivasan et al., 2015; Wei et al., 2023). It should be noted that TEER is influenced by the applied voltage and does not provide information regarding the transcellular transport of charged compounds (Hajal et al., 2021). Barrier permeability to solutes and overall barrier tightness is typically evaluated via tracer diffusion assays (e.g., FITC-dextran) (Bednarek et al., 2022), while live/dead imaging confirms cell viability and morphology is consistent with native BBB architecture (Bikmulina et al., 2022). Achieving native-like cellular morphologies is key to developing an accurate BBB model, as it reflects successful recapitulation of the physiological environment.

## 4 Applications of 3D bioprinting in modelling BBB in NDD research

The aetiologies of NDD involve intricate cross-talk between dysfunctional BMEC, PC, AC and neurons. 3D bioprinting, which allows precise, spatially controlled deposition of bioinks and multiple cell types, facilitates the creation of these complex cellular ecosystems, making it highly valuable in NDD research. Moreover, the incorporation of induced pluripotent stem cells (iPSCs) allows for the generation of humanised and patient-specific BBB models, thereby eliminating interspecies differences (Brown et al., 2019; Ito K.et al., 2011) and enabling personalised investigations into NDD pathophysiology and therapeutic response (Pérez-López et al., 2023).

Techniques such as TPL and DLP have been used to construct microvascular structures that mimic the native BBB microenvironment. Marino and colleagues employed a 3D BBB model by incorporating bEND.3 EC and U87 glioblastoma cells within microtubes of approximately 10 µm in diameter using the TPL technique (Marino et al., 2018). The bEnd.3 cells efficiently covered the tubular structures and the model demonstrated key BBB features, including TJ maturation (confirmed by ZO-1 IF) and barrier integrity (assessed via dextran diffusion), thus providing a powerful platform for high-throughput drug screening across the BBB. However, the absence of AC and PC in this biohybrid system resulted in a TEER of 75 ± 2 Ω cm^2^, substantially lower than *in vivo* values, limiting its suitability for studying NDD.

To address this limitation, Galpayage Dona et al. developed a more comprehensive model using DLP bioprinting that incorporated all major BBB cell types, AC, PC, and EC (Galpayage Dona et al., 2023). In this model, vascular structures were continuously perfused to activate mechanotransduction pathways and promote maturation. Treatment with Tumor necrosis factor alpha (TNF-α), known to decrease barrier tightness, significantly increased dextran leakage, confirming the model’s responsiveness to neuroinflammation, a common hallmark of NDD. To better simulate neuroinflammation, pro-inflammatory cytokines (e.g., TNF-α, IL-1β) or lipopolysaccharide have been introduced into the vascular lumen of bioprinted BBB models, enabling real-time assessment of barrier breakdown, upregulation of adhesion molecules (e.g., vascular cell adhesion molecule-1 (VCAM-1)), and immune cell adhesion and transmigration (Knox et al., 2022; Wei et al., 2023).

It is worth to notice that interleukin-6 (IL-6), elevated in AD and linked to BBB disruption (Lyra et al., 2021; Wu et al., 2015), has not yet been employed in 3D bioprinted BBB studies. IL-6, released by activated AC, triggers signal transducer and activator of transcription 3 (STAT3) pathways in AC and EC, inducing matrix metalloproteinase-9 (MMP-9) and vascular endothelial growth factor (VEGF) expression, leading to degradation of TJ proteins such as claudin-5, occludin, and ZO-1, thereby increasing BBB permeability (Gryka-Marton et al., 2025; Hu et al., 2025; Rose-John, 2017). This pathway is strongly associated with vascular dysfunction (Rose-John, 2017; Yang et al., 2022). Hence, targeting IL-6/STAT3 signalling to restore BBB function in diseased AC and EC may offer an effective strategy for developing novel therapeutics against NDD.

Beyond investigating inflammatory effects, 3D bioprinted BBB model also hold promise for evaluating the impact of natural compounds and virus infections in NDD pathogenesis (Abdelsalam et al., 2023; Kumar et al., 2018; Yousif et al., 2021). Similarly, 3D bioprinted models can be exposed to Aβ-induced toxicity to simulate AD (Yue et al., 2020) or subjected to oxygen-glucose deprivation to mimic ischemic stroke, a major risk factor for vascular dementia and other NDD, allowing studies on oxidative stress and reperfusion injury on BBB in a human context (Cho et al., 2015).

To further enhance DLP-printed model fidelity and matrix-cell interactions, Paone et al. created a tuneable, perfusable BBB model using DLP-printed PEG-norbornene hydrogels functionalised with HAVDI/IKVAV peptides, which promoted endothelial adhesion and TJ formation, as confirmed by ZO-1 staining, dextran permeability assays, and live/dead cell assays (Paone et al., 2024). Nonetheless, the current limitation for DLP-printed BBB model is the low resolution of polymerized layers, preventing is achievement of capillary-scale lumens.

Genetic mutations have also been incorporated into 3D bioprinting approaches to establish NDD models that exhibiting disease hallmarks. By transducing amyloid precursor protein (APP) genes with familial AD mutations to neural stem cells (NSC), Zhang and colleagues used 3D bioprinting technology to create a coaxial core-shell structure comprising a high-density cell suspension and Matrigel in the core, surrounded by alginate in the shell (Yi Zhang et al., 2022). This 3D printed AD model displayed superior self-assembly, extended cell survival, more complex metabolic activity, and differentiation rich in Aβ, highlighting 3D bioprinting as a promising tool for studying AD pathology and developing therapeutics. More recently, Royse et al. used stereolithography to bioprint a BBB model incorporating all key cell types expressing exogenous low-density lipoprotein receptor related protein 1 (LRP1) for studies of Aβ clearance, enabling mechanistic and pharmacological investigations modelling AD in NDD contexts (Royse et al., 2024).

Collectively, these studies demonstrate that 3D bioprinting enables the generation of biomimetic, multicellular, and perfusable BBB models that recapitulate key pathological features of NDD (Table 3), making them suited for advancing our understanding of BBB dysfunction, high-throughput drug screening and developing effective novel therapeutics for NDD.

**TABLE 3:** Compilation of 3D bioprinted BBB models applied in NDD studies. VE-cadherin: vascular endothelial cadherin; BCRP: breast cancer resistance protein; TfR: transferrin receptor.

Cell lines	Printing method	Barrier function characterisation	References
bEND.3 EC	LAP – TPL	ZO-1 staining, dextran permeability, TEER	Marino et al. (2018)
Human AC, BMEC, PC	LAP – DLP	ZO-1 staining, dextran permeability	Galpayage Dona et al. (2023)
Human AC, BMEC	LAP – DLP	ZO-1 staining, dextran permeability, ACviability	Paone et al. (2024)
Human AC, BMEC, PC	LAP – stereolithography	VE-cadherin/TfR/LRP1/BRCP staining, dextran permeability	Royse et al. (2024)

## 5 Conclusion and future perspectives

3D bioprinting enables precise spatial control in BBB models, creating perfusable, physiologically relevant structures through layer-by-layer deposition of biomaterials. By integrating multiple BBB cell types with brain-specific ECM components under digital design, it offers a reproducible platform for *in vitro* studies. However, challenges remain, including the lack of bioinks that mimic brain ECM, limited sub-capillary resolution, and immature tissue phenotypes.

Future progress will likely stem from combining bioprinted vascular networks with microfluidic systems and incorporating iPSC-derived cells into dynamic platforms for higher-throughput screening. Advances in BBB-specific bioinks and high-resolution printing will be key to producing reproducible, human-relevant BBB constructs for NDD modelling and CNS drug discovery.
